# 'What script am I meant to use?': a qualitative study in chronic primary pain

**DOI:** 10.3399/BJGPO.2024.0101

**Published:** 2024-12-11

**Authors:** Niamh Blythe, Carmel Hughes, Nigel D Hart

**Affiliations:** 1 School of Medicine, Dentistry and Biomedical Sciences, Queen’s University Belfast, Belfast, UK; 2 School of Pharmacy, Queen’s University Belfast, Belfast, UK

**Keywords:** general practitioners, healthcare guidelines, chronic primary pain, chronic pain, qualitative research

## Abstract

**Background:**

Chronic primary pain (CPP) as a diagnosis has been introduced in the recent International Classification of Diseases, 11^th^ Revision (ICD-11). CPP captures the *experience* of pain as the primary problem, without an underlying attributable cause. Dissemination of UK guidance regarding CPP represents the first time it has been recognised as a condition in its own right. Little is known about GP views concerning caring for patients with CPP, and how related guidance is viewed and applied in practice.

**Aim:**

To explore GP perspectives in relation to caring for people with CPP, including challenges encountered and use of related guidelines in practice.

**Design & setting:**

A UK-wide qualitative interview study in primary care.

**Method:**

Purposive and snowball sampling were used to recruit 15 GP participants from England, Northern Ireland, Wales, and Scotland. Semi-structured interviews were undertaken and analysed using reflexive thematic analysis.

**Results:**

The following three main themes were generated: (1) 'How to start? Problematic beginnings', which referred to difficulties regarding diagnosis; (2) 'Where to go? Mapping the management challenge'; and (3) 'How to get there? Navigating strategies and response', which explored GP awareness and acceptability of UK guidelines for chronic pain. Areas identified for potential improvement included increased access to non-pharmacological management (NPM) and secondary care services, support with deprescribing, and an expanded multidisciplinary team input.

**Conclusion:**

CPP is complex to both diagnose and manage. Although guidelines provide a useful framework, they pose challenges when translating into day-to-day practice.

## How this fits in

Chronic pain affects up to half of adults in the UK, with approximately 70% managed in the primary care setting. Chronic primary pain (CPP) represents a new diagnostic entity and has been incorporated into recent UK guidance. This study provides unique insights into GP perspectives when caring for people with CPP, and novel exploration into related guideline use. Our research highlights the difficulties faced in CPP diagnosis, management, and navigating appropriate strategies and response.

## Introduction

Pain is defined as chronic when persisting or recurring beyond 3 months.^1^ Between one-third to 50% of the UK population is affected by chronic pain, a figure expected to rise consistent with an ageing population.^2^ Chronic pain represents a substantial global health challenge, reported as the main cause of disability and disease burden worldwide by the Global Burden of Disease Study.^3^ More people are affected by chronic pain than diabetes, heart disease, and cancer collectively.^4^

**Figure 1.**
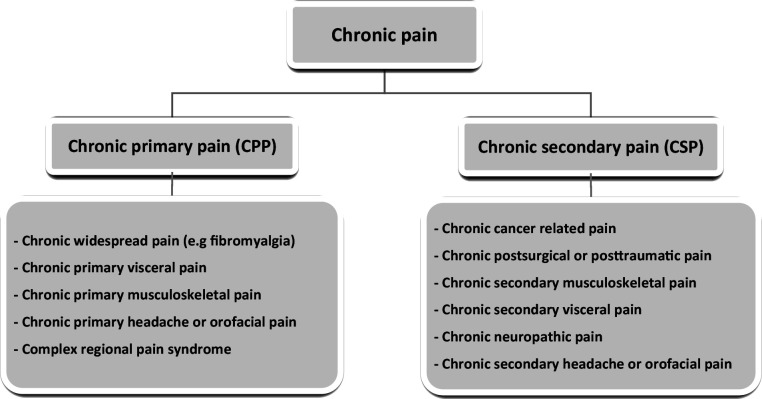
ICD-11 classification: chronic pain. Chronic primary pain (CPP) diagnoses differentiated from chronic secondary pain (CSP) diagnoses, which have known aetiology or established pathophysiology. An entity can span more than one diagnosis.^6,45^

Chronic primary pain (CPP) is a new diagnostic conceptualisation and is defined in the absence of an underlying attributable condition (Figure 1).^5,6^ The International Classification of Diseases, 11th Revision (ICD-11) came into effect in 2022, and includes a systematic coding system for chronic pain, introducing CPP as a diagnostic entity.^7^

In April 2021, the National Institute for Health and Care Excellence (NICE) published guidance (NG193) for the assessment of chronic pain and management of CPP.^5^ This provides clinical practice guidance for the UK, apart from Scotland where the Scottish Intercollegiate Guidelines Network (SIGN) guidelines for management of chronic pain are used (SIGN 136).^8^ NG193 promotes non-pharmacological management (NPM) with a move away from standard analgesic medications including opioids and gabapentinoids. SIGN 136 also encourages NPM but is not consistent with NG193 in that opioid medication may be considered in the short to medium term in specific circumstances.^9^

NG193 has received a mixed response from clinicians and pain experts, with concerns raised regarding the potential for patients to come to unintended harm and calls for revision of the guideline.^9–11^ A 'one-size-fits-all' approach to CPP management has potential to overlook individual needs,^10^ and reduce options available for management.^9^

To better understand the difficulties in provision of effective CPP management within primary care, this study aimed to explore GP perspectives in relation to caring for people with CPP, including the challenges encountered. We sought to explore knowledge, awareness, and acceptability of UK guidance for chronic pain.

## Method

### Recruitment

GPs working in the UK were eligible to participate in this semi-structured interview study. Invitations to express interest were disseminated by email to the Royal College of General Practitioners (RCGP) and via social media networks. Advertisements were shared in communications from WiseGP and PRIME Centre Wales, organisations promoting primary care scholarship and research. GPs who expressed interest were purposively sampled to provide a maximum-variation sample with regard to characteristics including age range, sex, UK jurisdiction, practice rurality, and deprivation. Snowball sampling was employed to aid recruitment whereby participants were asked to inform colleagues about the study.

From those who expressed interest, a purposive sample of GPs were emailed a participant information sheet and consent form. Following consent, online interviews were scheduled in line with participant availability. GPs who expressed interest, but were not included in the purposive sample, were thanked and provided with an explanation regarding the high response rate.

### Data collection

The interview topic guide (Supplementary File S1) was developed by the research team, using insights from published literature and professional knowledge pertaining to CPP and primary care. A patient public involvement (PPI) group was established specifically for this study, recruiting members through Versus Arthritis, Hope 4 ME and Fibromyalgia, and PIER NI (Public Involvement Enhancing Research).^12–14^ Input from PPI collaborators provided valuable insights and aided development and refinement of the topic guide. Pilot interviews with academic colleagues assisted in verifying appropriateness of interview content, clarity and coherence of questioning, and anticipated duration. Discussion focused on diagnostic and management challenges, and guideline use and implementation. The topic guide provided a framework and was used flexibly to facilitate more detailed enquiry.

Semi-structured interviews were undertaken by researcher NB and recorded using Microsoft Teams. Interviews continued until data sufficiency (the point at which incoming data generated no new properties or information to further address the research question) was achieved at 15 participants, as per consensus between the research team.^15^ From this point on no further GPs were consented. All participants received a £25 voucher to compensate for time and inconvenience.

### Data analysis

Interviews were recorded and transcribed verbatim by researcher NB. Data were analysed using Braun and Clarke’s six-phase approach to reflexive thematic analysis.^16^ Data familiarisation continued via re-reading transcripts and note-taking. Initial code generation was undertaken by NB with subsequent grouping of data and sub-theme creation. Three transcripts were chosen randomly and coded independently by researchers NB, CH, and NH, allowing for code review and refinement. The research team collaboratively identified and defined themes generated within the data on an inductive basis.

## Results

Of the 62 GPs who expressed interest, a purposive sample (ensuring maximum variation) of 44 were invited to participate in the study, with 16 subsequently agreeing to be interviewed and providing consent. Eighteen GPs who had expressed interest were informed that they were not needed as they did not fit the purposive sampling characteristics required. Interviews took place between August and November 2023, and lasted between 30 and 62 minutes (median 43). Fifteen GPs participated in an interview, while one could not be contacted despite consenting. Participant demographics are presented in Table 1. There was equitable distribution regarding sex, UK jurisdiction, and years since GP qualification. Most participants worked in areas of high or mixed deprivation, and in an urban setting as reported by participants.

**Table 1.** GP participant characteristics (*n* = 15)

Characteristic	*n (%*)
**Sex**	
Female	6 (40)
Male	9 (60)
**Practice UK jurisdiction**	
England	5 (33)
Ireland	4 (27)
Scotland	3 (20)
Wales	3 (20)
**Age range, years**	
25–34	3 (20)
35–44	8 (53)
45–54	3 (20)
55–64	1 (7)
**Years qualified as GP**	
<5	4 (27)
5–10	6 (40)
11–20	2 (13)
21–30	3 (20)
**Practice rurality**	
Rural	3 (20)
Urban	12 (80)
**Level of deprivation**	
High	8 (53)
Low	1 (7)
Mixed	6 (40)

Three key themes were generated using thematic analysis regarding GP perspectives caring for patients with CPP. Theme 1: 'How to start? Problematic beginnings', referred to diagnostic challenge; theme 2: 'Where to go? Mapping the management challenge', described issues including access to NPM, discontinuity of care, and de-prescribing; and theme 3: 'How to get there? Navigating strategies and response', explored GP use of UK guidance for chronic pain.

### Theme 1: How to start? Problematic beginnings

Most participants reported challenges regarding caring for patients with CPP began with difficulties surrounding diagnosis. Short consultation slots added diagnostic difficulty with the consensus '*10 minutes is never enough*' (Participant 1). Diagnosing CPP took place over multiple consultations, requiring a long time, owing to the multifactorial and complex nature of pain, as well as the need to rule out secondary causes:

*'The point of reaching the stage where you say, "well, there’s no underlying pathology that can be demonstrated here", takes place over months or years, especially with waiting lists.* […] *you're treating pain as a symptom long before you actually come to the realisation that it is a* […] *chronic pain syndrome …*' (Participant 13)

Time as a sub-theme was further stressed by 'in the meantime management'. Many described starting analgesia while awaiting diagnostic confirmation of CPP leading to future deprescribing difficulty:

*'… a huge challenge is the acute prescription becoming a chronic prescription, I think it’s massive.*' (Participant 4)

Some expressed a lack of confidence diagnosing CPP in primary care with a reliance on secondary care confirmation. Concerns involved dealing with uncertainty, potential medicolegal repercussions and fear of missing something:

*'I would find it really difficult in primary care just to make a diagnosis without actually having referred to secondary care.* […] *a rheumatologist who would be able to say, "*[…] *we ruled everything else out".*' (Participant 1)

Many reported interface difficulties accessing secondary care services for diagnostic support, leading to further time delay, describing CPP as a condition most often diagnosed and managed in primary care:

*'Rheumatologists don't want to be diagnosing patients with fibromyalgia. And so it’s* […] *get on and make the diagnosis and then think about what we can offer.*' (Participant 9)

Communicating the diagnosis to patients and providing a 'label' elicited mixed views. Some felt making a clear diagnosis helped to validate the experience of pain; others thought it unhelpful and were reluctant to provide formal diagnosis:

*'… patients find a diagnosis helpful because they feel that then they have an explanation for how they're feeling and that I think that’s therapeutically helpful …*' (Participant 12)

*'We just talk about pain because I'm not sure the label is helpful* […] *and could be misinterpreted that chronic means you can do nothing about it. I'm a bit careful about that.'* (Participant 11)

Most participants stressed the importance of effective communication and developing the doctor–patient relationship. There was an inherent awareness of associated stigma and past negative patient experiences when consulting clinicians for pain:

*'A feeling of being let down by health care. Not being able to explain their pain or provide a cure or a management strategy in line with their expectations. So frustration. Perhaps feeling of not being listened to, being fobbed off.*' (Participant 8)

*'… they* [patients] *tell me "I'm a bit hesitant in telling you because you think it’s all in my head". So the stigma, the taboo, the people looking down at them …'* (Participant 2)

### Theme 2: Where to go? Mapping the management challenge

The multifactorial and subjective nature of pain added to the complexity of CPP management, which was complicated further by the presence of comorbidity or co-existent mood disorder. Co-existent CSP, such as osteoarthritis, complicated prescribing approaches:

*' … people are just really struggling for lots of underlying reasons. But I think to try and really get to the bottom of it* […] *you'd have to deal with the whole backlog of trauma …*' (Participant 10)

*'… it can sometimes be a little bit difficult to differentiate them* [...]. *If they've got osteoarthritis* [...] *and you're giving them something for that. Well, it’s really hard to know if that’s treating any of the primary pain.*' (Participant 4)

CPP was viewed by participants as a difficult condition to treat, involving challenging consultations, with both patient and doctor expectations of management often going unmet:

*'People expect you as the doctor, to be able to deal with their pain, because it’s the cardinal medical complaint.*' (Participant 7)

*'My own expectations because* [...] *people only seek us out when they're in, having a dire problem. So my expectations,* […] *I am expected to fix it.'* (Participant 9)

Most participants were aware of the potential harm from certain prescribed analgesics, recognising that there were few other management options, which reinforced the challenges in prescribing:

*'If they've tried it* [opioids] *and it’s worked. It’s very, very difficult to* […] *then say, "I know it did and I told you it did, but that’s it. Tough luck. Finished."* […]*, because it almost feels inhumane.*' (Participant 4)

*'What I meant to do? What am I literally meant to say to the patient? What script am I meant to use?*' (Participant 2)

Difficulties in accessing NPM options were commonplace. Some participants were uncertain of the success and evidence base for such strategies. Many described a need for patient engagement and commitment before employing a NPM approach:

*'Certainly on the health service, seems like trying to fit* […] *a camel through the eye of a needle. There’s so many people could benefit from it* [psychological therapy] *if it was available.*' (Participant 3)

There was a lack of familiarity using medications, such as selective serotonin reuptake inhibitors (SSRIs) for CPP, with a tendency to reserve use for patients with comorbid mood disorder only. Patients may be at the ceiling of an antidepressant dose for a pre-existent mental health indication. Using antidepressants in the context of CPP required patient engagement and education owing to association with mental illness:

*'… convincing people that no, you're using them* [antidepressants] *to manage their pain and not because you think that they have a mental health disorder,* [...] *it can be challenging. I've definitely had push back from that before.*' (Participant 6)

Changing ways of working as a GP and discontinuity of care impeded timely follow-up. GPs working in a sessional capacity were less aware of local resources available:

*'… you're not there regularly and you lose them to follow-up and you don't know* [...] *whether or not they followed through the treatment plan that you that you laid out for them.*' (Participant 7)

*'… knowing what clinics, what support groups, or* […] *other health professionals are available in the area and how you refer to them,* […] *is really challenging and something you probably build up over time.*' (Participant 6)

GP heterogeneity in approach to management of CPP and deprescribing were shown to be a source of frustration to both patients and doctors:

*'I think if the culture and the practice is that medications are given out fairly freely, that sort of filters through to everybody working there* […]*, if you're the one who doesn't increase it and then they just go to another doctor who just straight away increases it, well then what’s the point …?*' (Participant 13)

Historic prescribing was viewed as more difficult to address than recent prescribing. Undertaking successful deprescribing required patient engagement and trust. Many reported choosing the *'right time'* to initiate deprescribing. Current NHS pressures were a further impediment to successful deprescribing:

*'It takes a long time to gradually and slowly down-titrate someone and needs good communication, lots of trust and continuity of care over a long time. And those are all elements that are being eroded by the systemic changes that are happening in the way that primary care is provided in the UK.*' (Participant 5)

*'As a clinician, my worry might be,* […] *they're on morphine* […]*. Some of these patients are actually worried about putting food on the table,* […] *it’s just making sure that we're picking the right time to start having that dialogue with them.*' (Participant 1)

### Theme 3: How to get there? Navigating strategies and response

Most participants were aware of either the NICE or SIGN guidelines for chronic pain, depending on jurisdiction of work. Many were familiar with the content of the respective guideline, but less than half actively used or consulted the guideline in day-to-day practice. NG193 provided a strong foundation and validation for proposed management strategies and led to an increased awareness of inappropriate prescribing. However, many of the same participants described the guidelines as idealistic and lacking pragmatism:

*'So aiming for that gold standard is always helpful but it’s always going to vary whether you get there or not because there’s so many different factors at play.*' (Participant 8)

*'I think sometimes* […] *it’s wishful thinking, isn't it? The idea of not using opioids, but I think it’s the right mentality to stress to us.*' (Participant 6)

*'Everything written down is very simple. OK what’s not on top of that is* […] *all that background story, all the emotion.* […] *if we could do everything off guidelines and didn't have to speak to anybody, it would all be great.*' (Participant 11)

Guideline use encouraged holistic management, with increased reluctance to prescribe standard analgesics and a feeling of empowerment to trial alternatives. Participants described an increased confidence using NPM and antidepressants for pain:

*'… my threshold for starting somebody on opioids has definitely much increased, that I would try really to be avoiding opioids* […] *and trying to explore non-pharmacological avenues.*' (Participant 15)

*'I am a lot more open to using SSRIs, and trying to explain to patients that," yeah, I don't think you're depressed.* […] *there is some evidence and it makes sense that these kinds of medications might help and that’s worth a try".'* (Participant 8)

GP awareness of guideline use as a steer rather than an inflexible approach prevailed, with emphasis on tailoring management to the individual and the need for patient collaboration.

*'… but actually when you bring patient preferences and patient expectations in, there could well be a compromise you have to come to.*' (Participant 4)

*'I know the kind of relationships I've got with my patients and* […] *what they will and will not accept. So I'll adjust these guidelines, which as you know, are not tramlines …*' (Participant 2)

Participants described inequity in accessing NPM services, including addition of multidisciplinary team (MDT) members in practice as well as disparity in service provision within localities and UK jurisdictions:

*'You just see the inverse care law left, right, and centre,* […] *trying to get some of the third sector things out of the big urban areas and to support our patients.*' (Participant 14)

*'… some practices obviously have counselling services and some of those are CBT* [cognitive behavioural therapy] *trained, although they're probably not pain CBT trained* […] *if resources were more equitably kind of available to all practices, that would make things easier.*' (Participant 13)

Some mentioned confusing terminology within NG193. The idea of fibromyalgia as a CPP syndrome was well-accepted, but conditions such as chronic primary headache were less well understood:

*'I don't tend to see people with* […] *chronic primary headache as much because a lot of those patients I see seem to be kind of more, let’s say migraine related. But I don't know if that if you're including those in it or if that’s a separate thing.*' (Participant 10)

NG193 has driven local innovation and response strategies to meet the challenge of CPP management. The importance of holistic care and MDT input was well accepted. Support for deprescribing included practice-based pharmacist involvement, local initiatives, and patient education:

*'They're* [practice based pharmacist] *absolutely integral. Particularly for a lot of the de-escalation or deprescribing follow-up*.' (Participant 7)

*'… we've been in the process of actually setting up a pain clinic to that would actually be not just dealing with the analgesia but actually looking at mental health.*' (Participant 1)

*'There are some very engaged patients who* […] *do actually appreciate the fact that you've taken the time to explain something that may not have been explained so well in the past.*' (Participant 9)

Proposals for future work and improvement included increased public health messaging and patient education. Some suggested physician education could be more inclusive of NPM:

*'And what about a national campaign for pain? So patients are educated?*' (Participant 2)

*'As GP trainees, we had talks on* […] *pain management and the analgesic ladder, but nothing ever about psychological therapies, so we can counsel our patients before.*' (Participant 6)

Although the majority of participants worked in a jurisdiction with NG194 as the standard, some mentioned confusion between the recommendations of NG193 and SIGN 136. Suggestions included further agreement between guidelines with respect to prescribing may be beneficial:

*'…for them* [guidelines] *to be like singing from the same hymn sheet, it would be helpful.'* (Participant 12)

## Discussion

### Summary

This study provides insights into the important challenges faced by GPs when diagnosing and managing CPP within primary care. Participants indicated the multifactorial, complex nature of CPP required time, appropriate resource, trusted doctor–patient relationships, and continuity of care to diagnose and adequately manage, all of which are lacking in today’s landscape of primary care.^17^ Difficulties accessing NPM options, and lack of familiarity with antidepressant use for CPP has led to problems with guideline implementation. GPs welcomed the guidelines’ holistic and person-centred approach, with support for further MDT involvement. However, inequity of access to MDT in practice was common and reinforced the sentiment of *'a lack of alternative options'* to standard analgesics.

### Strengths and limitations

This UK-wide study provides a comprehensive overview and unique insight into challenges of CPP management and application of related UK guidelines. PPI collaboration from conception to dissemination was pivotal to the success and relevance of the study. Data analysis involved independent coding of select transcripts by three researchers. Themes were collaboratively identified via a consensus approach and in accordance with the Consolidated criteria for reporting qualitative research (COREQ) checklist,^18^ thus reinforcing trustworthiness.

Qualitative research seeks to afford in-depth insight rather than claiming representativeness of the research findings. Reflecting on the context for this work (GPs working in UK primary care) should allow readers to determine the extent to which findings are theoretically transferable to their own settings. Study participants were self-selected and may have had pre-existing concerns or interest regarding CPP, meaning views may not be wholly representative of the wider profession. Urban and high deprivation practices were over-represented in our study sample, hence some findings may reflect issues encountered more frequently within these communities.

### Comparison with existing literature

The goal of this research was to provide a rich understanding of GP perspectives about care for patients with CPP. Our study highlights CPP as a condition for which primary care has an important management role.^19–22^ Unpacking the multifactorial complexity of CPP in a time-constrained health system presents diagnostic difficulty and frustration.^23–25^ Presence of comorbid conditions, including mood disorder, adds further challenge.^26^ With 25% higher psychiatric morbidity than the rest of the UK, this was notable among participants based in Northern Ireland, partly attributable to historical civil conflict.^27^ Consistent with our findings, NG193 reports CPP and CSP can co-exist,^5^ complicating both diagnosis and management given potential use of multiple guidelines, which may have contradictory recommendations.

A recent Australian study highlighted the need for GP education and consistency of approach when educating patients about chronic pain and setting realistic management expectations.^28^ Our findings support conclusions indicating patient expectations often go unmet, adding to frustration for patient and GP alike.^21,25,29,30^ Heterogeneity in approach to prescribing for CPP and use of guidelines is a further barrier to effective management.^28,31^

Continuity of care is a core principle of general practice and associated with greater satisfaction rates, better healthcare outcomes, and improved cost-effectiveness.^17,32^ GPs in this study recognised continuity of care as key when delivering appropriate CPP management, follow-up, and deprescribing.^33^ Changing ways of working and patterns of provision impact the capacity to provide continuity in primary care.^32^ GPs reported how knowledge regarding local resources and referral options accumulated with time, which is often difficult to achieve in a sessional capacity.

A lack of congruity between guideline recommendations and NPM access was voiced by participants as a major influencer of prescribing practices,^31^ echoing Finestone and colleagues' view that *'opioid prescribing is a surrogate for inadequate pain management resources'.* ^34^ A UK study by Zambelli and colleagues set a comparative baseline for the efficacy of NG193, with authors concluding service provision of psychological therapies needed to increase to meet NG193 recommendations.^35^ A 2023 study, exploring the experience of primary care advanced nurse practitioners (ANPs) and opioid deprescribing, reported lack of availability of NPM alternatives for managing CPP as a barrier to deprescribing and NG193 implementation.^36^ As medication use is prevalent among chronic pain populations, special provision is needed within primary care to allow for medication review.^35^ GPs in our study emphasised the value of the MDT in provision of holistic person-centred care, in particular the positive impact on healthcare delivery of practice-based pharmacists, who are ideally placed to recognise and address prescribing-related issues.^36–38^

### Implications for practice

Clinical guidelines are not rigid rules but valuable decision-making support tools. This study highlighted GPs appreciated NG193 and SIGN 136 promoted a patient-centred approach, encouraging holistic care. The importance of MDT, NPM access, and support with deprescribing was recognised, placing emphasis on patient and clinician needs. However, the results indicate that availability of chronic pain guidelines alone may not be sufficient to ensure a shift in clinician prescribing practices.^39,40^

Dilemmas faced by clinicians, as articulated in this study, relate to diagnostic ambiguity, clinical heterogeneity and securing an elusive outcome owing to the subjective nature of pain. This complexity renders a lack of a straightforward solution with new aspects of the problem continually revealed as evaluation continues, in essence, a 'wicked problem'.^41^ First discussed by Rittel and Webber in 1973, the concept of 'wicked problems' refers to defiant, complex, and recurrent issues, often with conflicting and changing requirements.^42^ Reconceptualisation of CPP through this lens may offer further understanding as to design meaningful interventions and present new insights when tackling this issue.^43,44^

The title for this article is a direct participant quote: *'What script am I meant to use?'* For the authors the ambiguity of the word '*script'* (potential meanings: 1. the structure and content of something said in a consultation [a ‘speech’] versus 2. shorthand for a doctor’s prescription [for medicine]) captured more than one of the inherent dilemmas experienced by GPs when seeking to meaningfully address the needs of people living with CPP. It may well be that clinicians would benefit from detailed training on how to structure what is said in consultations related to CPP.

## Data

The dataset for this study cannot be made available due to the possibility of identification of participants who had been informed that they would not be identified in any way, and gave their consent to participate on this basis.

## References

[1] International Association for the Study of Pain (IASP) (2024). Definitions of chronic pain syndromes.

[2] Fayaz A, Croft P, Langford RM (2016). Prevalence of chronic pain in the UK: a systematic review and meta-analysis of population studies. BMJ Open.

[3] Vos T, Abajobir AA, Abbafati C (2017). Global, regional, and national incidence, prevalence, and years lived with disability for 328 diseases and injuries for 195 countries, 1990–2016: a systematic analysis for the Global Burden of Disease Study 2016. Lancet.

[4] Tsang A, Von Korff M, Lee S (2008). Common chronic pain conditions in developed and developing countries: gender and age differences and comorbidity with depression-anxiety disorders. J Pain.

[5] National Institute for Health and Care Excellence (NICE) (2021). Chronic pain (primary and secondary) in over 16s: assessment of all chronic pain and management of chronic primary pain.

[6] Nicholas M, Vlaeyen JWS, Rief W (2019). The IASP classification of chronic pain for ICD-11: chronic primary pain. Pain.

[7] Raja SN, Carr DB, Cohen M (2020). The revised International Association for the Study of Pain definition of pain: concepts, challenges, and compromises. Pain.

[8] Healthcare Improvement Scotland (2019). SIGN 136: management of chronic pain: a national clinical guideline.

[9] Smith BH, Colvin LA, Donaldson-Bruce A, Birt A (2021). Drugs for chronic pain: we still need them. Br J Gen Pract.

[10] Korwisi B, Barke A, Kharko A (2021). Not really nice: a commentary on the recent version of nice guidelines [NG193: chronic pain (primary and secondary) in over 16s: assessment of all chronic pain and management of chronic primary pain] by the Pain Net. Pain Rep.

[11] Kmietowicz Z (2021). Doctors raise concerns about NICE guidelines on chronic primary pain. BMJ.

[12] Versus Arthritis (2024). Patient and Public Involvement and Engagement in Research.

[13] Hope 4 ME and Fibromyalgia (2024). Homepage. https://hope4mefibro.org/.

[14] Public Health Agency (2024). PIER NI (Public Involvement Enhancing Research).

[15] LaDonna KA, Artino AR, Balmer DF (2021). Beyond the guise of saturation: rigor and qualitative interview data. J Grad Med Educ.

[16] Braun V, Clarke V (2006). Using thematic analysis in psychology. Qual Res Psychol.

[17] Haggerty JL, Reid RJ, Freeman GK (2003). Continuity of care: a multidisciplinary review. BMJ.

[18] Tong A, Sainsbury P, Craig J (2007). Consolidated criteria for reporting qualitative research (COREQ): a 32-item checklist for interviews and focus groups. Int J Qual Health Care.

[19] Breivik H, Collett B, Ventafridda V (2006). Survey of chronic pain in Europe: prevalence, impact on daily life, and treatment. Eur J Pain.

[20] Upshur CC, Luckmann RS, Savageau JA (2006). Primary care provider concerns about management of chronic pain in community clinic populations. J Gen Intern Med.

[21] Stannard C, Johnson M (2003). Chronic pain management—can we do better? An interview-based survey in primary care. Curr Med Res Opin.

[22] Hasselström J, Liu-Palmgren J, Rasjö-Wrååk G (2002). Prevalence of pain in general practice. Eur J Pain.

[23] Ng W, Slater H, Starcevich C (2021). Barriers and enablers influencing healthcare professionals’ adoption of a biopsychosocial approach to musculoskeletal pain: a systematic review and qualitative evidence synthesis. Pain.

[24] Goodwin J, Kirkland S (2021). Barriers and facilitators encountered by family physicians prescribing opioids for chronic non-cancer pain: a qualitative study. Health Promot Chronic Dis Prev Can.

[25] Gill S, Bailey J, Nafees S, Poole R (2022). A qualitative interview study of GPs’ experiences of prescribing opioid medication for chronic pain. BJGP Open.

[26] Bruggink L, Hayes C, Lawrence G (2019). Chronic pain: overlap and specificity in multimorbidity management. Aust J Gen Pract.

[27] Rahman A, Kane J, Montastruc F, Renoux C (2021). Trends in new prescription of gabapentinoids and of coprescription with opioids in the 4 nations of the UK, 1993–2017. Br J Clin Pharmacol.

[28] Gilkes L, Bulsara C, Mavaddat N (2023). Chronic non-cancer pain management — insights from Australian general practitioners: a qualitative descriptive study. Aust J Prim Health.

[29] Geurts JW, Willems PC, Lockwood C (2017). Patient expectations for management of chronic non-cancer pain: a systematic review. Health Expect.

[30] Stannard C, Bernstein I (2021). NICE guideline NG193 for chronic pain: reasons to be cheerful. Br J Gen Pract.

[31] Desveaux L, Saragosa M, Kithulegoda N, Ivers NM (2019). Understanding the behavioural determinants of opioid prescribing among family physicians: a qualitative study. BMC Fam Pract.

[32] Jeffers H, Baker M (2016). Continuity of care: still important in modern-day general practice. Br J Gen Pract.

[33] Kang Y, Trewern L, Jackman J (2023). Chronic pain: definitions and diagnosis. BMJ.

[34] Finestone HM, Juurlink DN, Power B (2016). Opioid prescribing is a surrogate for inadequate pain management resources. Can Fam Physician.

[35] Zambelli Z, Halstead EJ, Iles R (2022). The 2021 NICE guidelines for assessment and management of chronic pain: a cross-sectional study mapping against a sample of 1,000* in the community. Br J Pain.

[36] McCann C, McCauley CO, Harkin D (2024). Barriers and facilitators to opioid deprescribing among advanced nurse practitioners: a qualitative interview study. J Adv Nurs.

[37] McDermott ME, Smith BH, Elliott AM (2006). The use of medication for chronic pain in primary care, and the potential for intervention by a practice-based pharmacist. Fam Pract.

[38] Hasan Ibrahim AS, Barry HE, Hughes CM (2023). GPs’ and pharmacists’ views of integrating pharmacists into general practices: a qualitative study. Br J Gen Pract.

[39] Victor TW, Alvarez NA, Gould E (2009). Opioid prescribing practices in chronic pain management: guidelines do not sufficiently influence clinical practice. J Pain.

[40] McCalmont JC, Jones KD, Bennett RM, Friend R (2018). Does familiarity with CDC guidelines, continuing education, and provider characteristics influence adherence to chronic pain management practices and opioid prescribing?. J Opioid Manag.

[41] Sinskey JL, Margolis RD, Vinson AE (2022). The wicked problem of physician well-being. Anesth Clin.

[42] Rittel HWJ, Webber MM (1973). Dilemmas in a general theory of planning. Pol Sci.

[43] Burns D, Hyde P, Killett A (2013). Wicked problems or wicked people? Reconceptualising institutional abuse. Sociol Health Illn.

[44] Termeer CJAM, Dewulf A, Biesbroek R (2019). A critical assessment of the wicked problem concept: relevance and usefulness for policy science and practice. Pol Soc.

[45] Treede R-D, Rief W, Barke A (2019). Chronic pain as a symptom or a disease: the IASP Classification of Chronic Pain for the International Classification of Diseases (ICD-11). Pain.

